# Supplementary material for: 3′UTR Deletion of NONO Leads to Corpus Callosum Anomaly, Left Ventricular Non-Compaction and Ebstein’s Anomaly in a Male Fetus
Source: Diagnostics (Basel). 2022 Sep 28;12(10):2354. doi: 10.3390/diagnostics12102354 (PMC9600144; doi:10.3390/diagnostics12102354)
Supplement: Supplementary file 1 [file diagnostics-12-02354-s001.zip › diagnostics-1888761-supplementary.pdf]

Supplementary methods

Primers Sequence for quantitative Real Time-PCR on *BCYRN1* gene performed as CNV segregation analysis

***BCYRN1*_FW** AGAGCTAAAATGCCCCCGAG

***BCYRN1*_RV** TCAATTCGTCTTTCCAGCCAA

***TERT*_FW** CTGTGACACTTCAGCCGCAA

***TERT*_RV** GAGGAACATGCGTCGCAAA

Primers Sequence for NONO expression analysis by quantitative Real Time-PCR on cDNA

***NONO*_ex12-13_Fw** TGGAACTTTGGGATTGACCC

***NONO*_ex12-13_Rev** GCCTGACCAAAGCGTTCAG

***POLR2A*_Fw** GGTGCAACCCCTGCCTATGG

***POLR2A*_Rv** TGGGGTCATTCCACTCCCAAC

Primers Sequence used for breakpoints refinement and Sanger Sequencing

***NONO*_3_Fw:** GAGGAAGGTCTGGAACAGCC

***NONO*_3_Rev:** CCACACTGGGCCCCAAATAA

***NONO*_4_Fw** TATTTTGCCAGTGACATCTGTTG

***NONO*_4_Rev** CTCCTACCTTTGGAACTATCTC

***NONO*_5_Fw** CATCATCTCTCATGATGGGAAC

***NONO*_5_Rev** TTGTTAATGCTCACTACCTATATC

***NONO*_6_Fw** GACTGAAGTTTCCTTACATGAG

***NONO*_6_Rev** GTTCTTTGCCATTCCAGTTTAG

***NONO*_seq4_Rev** GGCTATCCCGAGATATCTTAC

***NONO*_seq5_Rev** CTGAAAAAGTATTGCTGGACATC

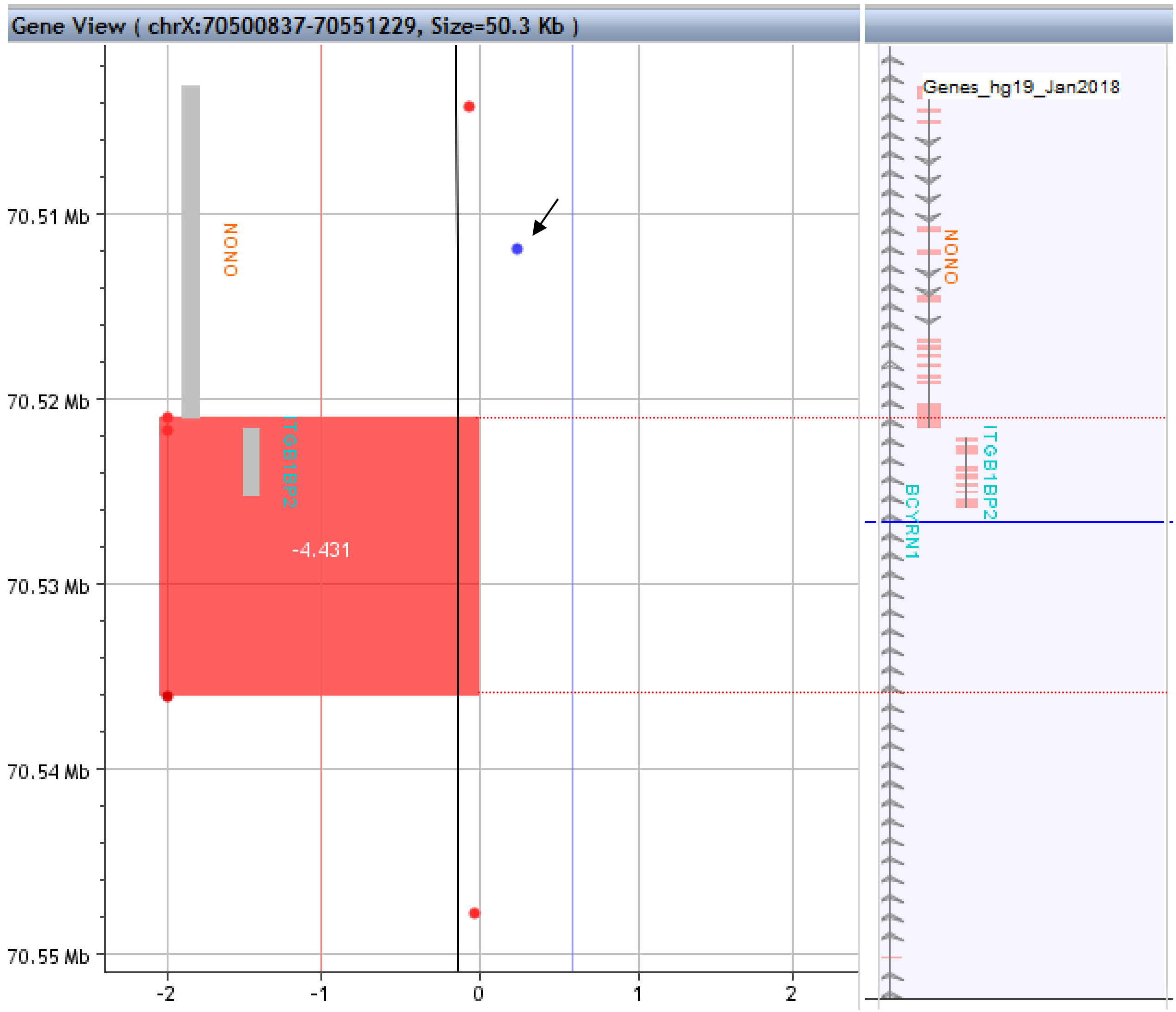

Figure S1: array-CGH analysis

A 15 Kb deletion (red area) at chromosome Xq13.1 detected by array-CGH analysis (180K; Agilent Technologies), called by three consecutive probes and involving the entire sequence of *ITGB1BP2,* an intronic sequence of *BCYRN1* (https://genome.ucsc.edu/;hg19. Last access on 28/04/2022) and the last part of the disease-gene *NONO* (red lines). The array-CGH analysis, due to the distribution of probes (see black arrow for the last non-deleted probe) did not allowed to establish exactly which part of this gene was deleted.
